# Association of migraines with brain tumors: a nationwide population-based study

**DOI:** 10.1186/s10194-018-0944-1

**Published:** 2018-11-15

**Authors:** Chao-Hung Chen, Jau-Jiuan Sheu, Yi-Chun Lin, Herng-Ching Lin

**Affiliations:** 1 Department of Thoracic Surgery, Mackay Memorial Hospital, Taipei, Taiwan; 2 Research Center of Sleep Medicine, College of Medicine, Taipei Medical University, Taipei, Taiwan; 3 Department of Neurology, Taipei Medical University Hospital, Taipei, Taiwan; 4 Department of Neurology, School of Medicine, College of Medicine, Taipei Medical University, Taipei, Taiwan; 5 Biostatistics Center, College of Management, Taipei Medical University, Taipei, Taiwan; 6 School of Health Care Administration, Taipei Medical University, Taipei, Taiwan; 7 Sleep Research Center, Taipei Medical University Hospital, Taipei, Taiwan

**Keywords:** Migraine, Brain tumor, Epidemiology

## Abstract

**Background:**

Several studies examined headaches as a symptom of brain neoplasms. Nevertheless, very few studies attempted to specifically evaluate the role of headaches as a risk factor. This study aimed to investigate the risk of migraine occurrence in the preceding years among patients diagnosed with brain tumors and unaffected controls.

**Methods:**

Data were obtained from the Taiwan National Health Insurance Research Database. In total, 11,325 adults with a first-time brain tumor diagnosis were included as cases, together with 11,325 unaffected matched controls. Each individual was traced in the healthcare claims dataset for a prior diagnosis of migraines. Conditional logistic regressions were performed to calculate the odds ratio (OR) and the corresponding 95% confidence interval (CI) to present the association between brain tumors and having previously been diagnosed with migraines.

**Results:**

We found that among patients with and those without brain tumors, 554 (4.89%) and 235 (2.08%) individuals, respectively, were identified as having a prior migraine diagnosis. Compared to unaffected controls, patients with brain tumors experienced an independent 2.45-fold increased risk of having a prior migraine diagnosis. The risks were even higher among men (odds ratio (OR) = 3.04, 95% confidence interval (CI) = 2.29~ 4.04) and after patients who had received a prior migraine diagnosis within 3 years were excluded (OR = 1.91, 95% CI = 1.59~ 2.29).

**Conclusions:**

This is the first report demonstrating the occurrence of brain tumors to be associated with a prior migraine history, for both men and women, in a population-based study.

## Introduction

Migraines, depicted as severe headache attacks, are a common neurological disorder affecting approximately 11.7% of the population [1–4]. The prevalence peaks in middle life, with more women (17.1%) than men (5.6%) suffering from this distressing condition. Migraines and tension-type headaches account for most patients with headache disorders, based upon the International Classification of Headache Disorders (ICHD) [2]. Migraines are associated with a lower health-related quality of life, more disability (e.g., an inability to work, attend social functions, and carry out routine chores), and greater healthcare utilization, thus causing substantial socioeconomic impacts [5–7].

Numerous neurological and medical comorbidities are frequently observed among patients with migraines, including obesity, sleep disorders, asthma, ischemic stroke, and cardiovascular diseases [8–10]. Although brain lesions are not commonly detected, patients with migraine may experience anxiety of having a more-serious underlying illness, such as a lethal condition of brain tumors. Such worry or apprehension associated with migraines may influence general practitioners’ assessments for referrals to neurological specialists [11]. This typically reflects the research and public concerns of the association between brain tumors and headaches in general, or migraines in particular.

Although brain neoplasms in headache patients are less common, headaches frequently occur (about 33%~ 71%) in patients with brain tumors [12–14]. Approximately 58.78% of patients with brain neoplasms also reported headaches in a 2-year prospective study, and half of them declared headaches to be their first complaint [15]. Among patients operated on for intracranial tumors in a prospective cohort study, 55% complained of headaches [16]. As headaches are the most common complaint and are frequently reported as the initial symptom among patients with malignant brain tumors, it was speculated that headaches or migraines should be treated as risk factors for the development of brain tumors or merely be considered as the “first sign” of brain neoplasms.

Several studies examined headaches as a symptom of brain neoplasms [12–15]. Nevertheless, only one population-based study conducted by Kurth et al. attempted to specifically evaluate the role of headaches as a risk factor [17]. That large, prospective cohort study found that a headache history was not associated with increased risks of subsequent brain neoplasm occurrence. Nevertheless, as was noted in their limitations section, headaches were self-reported with possible misclassification. Utilizing a specific study population of female health professionals aged 45 years and older further restricted the generalizability of the findings to other settings, especially men. Further investigation of the role of migraines in the development of brain rumors and clarification to advance clinical management for patients with migraines were thus deemed critical.

Thus, our nationwide, population-based nested case-control study was carried out to investigate the risks of migraine occurrence in preceding years among patients diagnosed with a brain tumor and unaffected matched controls. Both men and women were examined for the risks of brain neoplasms associated with a prior migraine history.

## Methods

### Database

We retrieved data on sampled patients from the Taiwan National Health Insurance (NHI) Research Database (NHIRD). The Taiwan’s NHI program is a single-payer program which was implemented in 1995. The NHIRD includes medical claims data for approximately 99% of the Taiwanese population (*n* = 23 million) under Taiwan’s NHI program. The NHIRD enables scientists in Taiwan to longitudinally follow-up medical services of all enrollees for research purposes. This study was approved by the institutional review board of Taipei Medical University (TMU-JIRB N201803087).

### Selection of cases and controls

As for selection of cases for this case-control study, we first identified 12,355 patients who had received a first-time diagnosis of a brain tumor (International Classification of Diseases, Ninth Revision, Clinical Modification (ICD-9-CM) code 191) between January 1, 2006 and December 31, 2013. We excluded 1012 patients aged less than 18 years. We further defined the date of receiving their first-time brain tumor diagnosis as the index date. In total, 11,325 patients with a brain tumor were included as cases in this study.

As for the controls, we likewise retrieved them from the Registry of beneficiaries of the NHIRD. In total, 11,325 controls (one control per case) were randomly selected and matched with cases in terms of sex, age, monthly income (NT$0~NT$15,840; NT$15,841~NT$25,000; ≥NT$25,001, the government-stipulated minimum wage for full-time employees in Taiwan was NT$15,840, and the average exchange rate in 2017 was US$1 ≈ New Taiwan (NT)$30), geographical location (northern, central, eastern, and southern Taiwan), urbanization level (five levels, with 1 being the most urbanized and 5 the least), and index year. While for cases, we assigned the year of the index date as the year in which the cases received their first brain tumor diagnosis, for controls, the year of the index date was simply a matched year in which controls had an ambulatory care visit. Furthermore, we indicated the first ambulatory care visit that occurred in the index year as the index date for controls.

### Exposure assessment

We identified migraine cases based on ICD-9-CM code 346. This study only included migraine cases if they had received at least two diagnoses of migraines prior to the index date since coding validity is often disputed for administrative databases. In addition, the included migraine cases had to have received at least one diagnosis of migraines from a certified neurologist.

### Statistical analysis

We used the SAS system (SAS System for Windows, vers. 8.2, SAS Institute, Cary, NC) for data analysis in this study. We used χ ^2^ tests to compare differences in monthly income, urbanization level, and geographic location between cases and controls. Furthermore, we used conditional logistic regression analyses (conditioned on sex, age, monthly income, geographical location, and urbanization level) to calculate the odds ratio (OR) and the corresponding 95% confidence interval (CI) to present the association between brain tumors and having previously been diagnosed with migraines. We used the conventional *p* ≤ 0.05 for statistical significance in this study.

## Results

Table 1 illustrates the distribution of sociodemographic characteristics of patients who did and those who did not have a brain tumor. The mean age of sampled patients was 57.1 years (standard deviation = 16.8 years). Pearson ^χ2^ tests showed that there was no significant difference between cases and controls in terms of the characteristics of sex, monthly income, geographical location, or urbanization level.

**Table 1:** Patients with brain tumors and controls -- sociodemographic characteristics (*n*=22,650)

Variable	Patients with a brain tumor *n* = 11,325		Controls *n* = 11,325		*p* value
	No.	%	No.	%	
Males	5966	52.7	5966	52.7	> 0.999
Age, mean (SD) (years)	57.1 (16.8)		57.1 (16.8)		> 0.999
Monthly income					> 0.999
< NT$15,841	5512	48.7	5512	48.7	
NT$15,841~ 25,000	3661	32.3	3661	32.3	
≥ NT$25,001	2152	19.0	2152	19.0	
Urbanization level					> 0.999
1 (most urbanized)	2962	26.2	2962	26.2	
2	2921	25.8	2921	25.8	
3	1693	15.0	1693	15.0	
4	1672	14.8	1672	14.8	
5 (least urbanized)	2077	18.3	2077	18.3	
Geographical region					> 0.999
Northern	5192	45.9	5192	45.9	
Central	3022	26.7	3022	26.7	
Southern	2867	25.3	2867	25.3	
Eastern	244	2.1	244	2.1	

*SD* standard deviation

The average exchange rate in 2017 was US$1 ≈ New Taiwan (NT) 30

Table 2 presents the distributions of prior migraines between cases and controls. It shows that there were significant differences between cases and controls in the prevalence of prior migraines (4.89% vs. 2.08%, *p* < 0.001). Furthermore, the conditional logistic regression analyses (conditioned on sex, age, monthly income, geographical location, and urbanization level) suggested that the odds of prior migraines was 2.45-times greater (95% CI = 2.09~ 2.96, *p* < 0.001) for patients with a brain tumor than for controls.

**Table 2:** Odds ratios (ORs) of prior migraines among patients with a brain tumor and the controls (*n* = 22,650)

Presence of prior migraines	Total sample *n* = 22,650		Patients with a brain tumor *n* = 11,325		Controls *n* = 11,325	
	No.	%	No.	%	No.	%
Yes	789	3.48	554	4.89	235	2.08
No	21,861	96.52	10,771	95.11	11,090	97.92
OR ^a^ (95% CI)	–		2.45*** (2.09~ 2.96)		1.00	

Notes: ^a^Conditional logistic regression conditioned on patient’s age, sex, monthly income, geographic region, and urbanization level; *** *p* < 0.001

*CI* confidence interval

Table 3 describes the sensitivity analysis. After excluding patients who had received a first-time migraine diagnosis within 1 year prior to the index date, we found a significant association still existed between brain tumors and migraines (OR = 1.99, 95% CI = 1.68~ 2.34). In addition, the association between brain tumors and migraines remained after excluding patients who had received a first-time migraine diagnosis within 2 (OR = 1.87, 95% CI = 1.57~ 2.23) or 3 years (OR = 1.91, 95% CI = 1.59~ 2.29) prior to the index date.

**Table 3:** Sensitivity analysis

Presence of prior migraines	Excluding patients who received a first-time migraine diagnosis within 1 year prior to the index date		Excluding patients who received a first-time migraine diagnosis within 2 years prior to the index date		Excluding patients who received a first-time migraine diagnosis within 3 years prior to the index date	
	Patients with a brain tumor *n* = 11,184	Controls *n* = 11,307	Patients with a brain tumor *n* = 11,136	Controls *n* = 11,294	Patients with a brain tumor *n* = 11,104	Controls *n* = 11,169
Yes	415 (3.71)	217 (1.92)	368 (3.30)	204 (1.81)	337 (3.03)	183 (1.62)
No	10,769 (96.29)	11,090 (98.08)	10,760 (97.57)	11,090 (98.19)	10,767 (96.97)	11,090 (99.29)
OR ^a^(95% CI)	1.99*** (1.68~ 2.34)	1.00	1.87*** (1.57~ 2.23)	1.00	1.91*** (1.59~ 2.29)	1.00

Notes: ^a^Conditional logistic regression conditioned on patient’s age, sex, monthly income, geographic region, and urbanization level; *** *p* < 0.001; *OR* odds ratio, *CI* confidence interval

Table 4 further shows the association between brain tumors and migraines according to sex. We found that there was a significant association between brain tumors and migraines regardless of sex. In particular, males patients with a brain tumor were as great as 3-times more likely to have received a diagnosis of prior migraines compared to their male controls (OR = 3.04, 95% CI = 2.29~ 4.04).

**Table 4:** Odds ratios (ORs) of prior migraines among patients with a brain tumor and the controls according to sex (*n* = 22,650)

Presence of prior migraines	Males		Females	
	Patients with a brain tumor *n* = 5966	Controls *n* = 5966	Patients with a brain tumor *n* = 11,028	Controls *n* = 11,181
Yes	193 (3.23)	65 (1.09)	361 (6.74)	170 (3.17)
No	5773 (96.77)	5901 (98.91)	4998 (93.26)	5189 (96.83)
OR ^a^ (95% CI)	3.04*** (2.29~ 4.04)	1.00	2.22*** (1.84~ 2.67)	1.00

Notes: ^a^Conditional logistic regression conditioned on patient’s age, monthly income, geographic region, and urbanization level; ***p < 0.001; *CI* confidence interval

## Discussion

Our study leads the way in examining the role of headaches as a potential risk factor for the development of malignant brain tumors, utilizing a population-based study design for both men and women. In this nested case-control study, we found that among patients with and those without brain tumors, 554 (4.89%) and 235 (2.08%) individuals, respectively, were identified as having a prior migraine diagnosis. Compared to unaffected controls, patients with brain tumors experienced a 2.45-fold increased risk of having a prior migraine diagnosis, after the factors of age, sex, monthly income, geographic region, and urbanization level were considered in the conditional logistic regression analyses. To address the possibility that migraines might merely present as a “first sign” of brain tumors, we further excluded patients with any migraine history within 1~ 3 years prior to the neoplasm diagnosis. The results slightly attenuated but remained significant that the ORs altered from 1.99 to 1.91 as patients who received a prior first-time migraine diagnosis within 1~ 3 years, respectively, were excluded. Finally, we observed even higher risks of brain tumors associated with a prior history of migraines among men (OR = 3.04, 95%CI = 2.29~ 4.04) than women (OR = 2.22, 95%CI = 1.84~ 2.67).

Earlier studies suggested that Asians have a lower migraine prevalence than Westerners, that further depends on case definitions and methodologies in assessments [18]. Several surveys conducted in Asia (e.g., Taiwan, Japan, China, and Malaysia) using International Headache Society (IHS) criteria reported the migraine prevalence to range 1%~ 9% [19–22], fairly consistent with the prevalence identified in our study (2.08% for those without brain tumors) using migraine healthcare utilization for case identification. Our estimate was lower than the reported prevalence in Asia by Woldeamanuel [23] (about 10.1%) but was slightly higher than a systematic review report by Stark et al. [24] (about 1.0–1.7%). Methodological issues may be involved for the differences, especially the sample recruited (e.g., population-, community-, or hospital- based), study design, and the diagnostic criteria/assessments adopted (e.g., telephone interview, face-to-face interview, or clinical evaluation based upon the IHS criteria [2]). Other factors may also worth considering. Ethnic differences in pain perception and response were observed [25]. Healthcare system features (e.g., access to medication and physician consultations) may also be pertinent. Especially in Chines culture, headache may be considered an emotional problem or weakness to possibly discourage reporting of symptoms and utilization of healthcare [26]. It should be further noted that brain neoplasms in headache patients are not common. In a large prospective UK study, the 1-year risk of a malignant brain tumor was 0.15%, increasing to 0.28% above the age of 50 years, among patients classified as having new undifferentiated headaches. For primary headaches, a risk of 0.045% was reported [27].

Despite this, the migraine-tumor association has garnered continued attention as approximately 33%~ 71% of patients with brain neoplasms presented the symptom of headaches [12–14]. Several studies suggested headaches to be an early indicator of central nervous system tumors [2, 16, 28, 29]. However, migraines may exist in a wide variety of circumstances, thus complicating the possibility of linking migraines to brain neoplasms. Although there are long-lasting speculation and concerns, very limited evidence, in fact only one large-scale population-based study conducted by Kurth et al., examined the possible role of migraines as a risk factor for the development of tumor neoplasms [17]. Contradicting the null findings proposed by Kurth et al., our study found the occurrence of brain neoplasms to be associated with a prior migraine diagnosis. Although the strength of the risks slightly dropped (from 2.45 to 1.91) but the significance remained (*p* < 0.001) if patients who received a first-time migraine diagnosis within 3 years prior to the brain tumor identification were excluded. It has been suspected that a migraine-like headache may herald brain tumor and; therefore, some “migraine” cases could conceal an initial tumor. Nevertheless, our results cannot rule out the possibility that the risks of brain neoplasms may be associated with prior migraine exposure, not simply presented headache as a “first-sign” of brain tumors. Variations in methodology may help explain differences in these findings. As was mentioned in Kurth et al.’s study, self-reported migraines with possible misclassification and few brain tumor cases (*n* = 52) during a follow-up time of 15.8 years may have reduced their power to detect a true difference. Indeed, the hazard ratio (HR) reported was slightly higher than 1 (HR = 1.18, 95% CI = 0.58~ 2.41), with a wide confidence interval, possibly due to the smaller event size, encompassing 1 [17].

Several methodological strengths of our study should be noted, including the use of a large-scale population-based study to evaluate the risks of brain tumors associated with a prior diagnosis of migraines to fairly exempt our study from selection and non-response biases. In using a claims dataset, migraines were diagnosed based upon IHS criteria by certified neurologists. In addition, this study was nested within a prospectively recorded claims dataset to relieve concerns of recall bias usually associated with a case-control study design. This nested case-control study was also deemed suitable for detecting rare events, as primary brain tumors have low incidence rates of approximately 8.5~ 14 per 100,000 person-years across regions [30–32]. The large sample size in our study (i.e., 11,325 patients with a brain tumor, together with 11,325 unaffected controls) may have yielded ample statistical power for statistical analyses.

In terms of sex differences, Kurth et al. specifically examined apparently healthy women only in a prospective study and reported a null finding of migraines being associated with subsequent brain tumor risks. In addition to this single population-based study investigating migraines as a potential risk factor for brain neoplasms, another study attempted to assess the risks of breast cancer among women, by examining the involvement of hormonal factors in both migraines and breast cancer [17]. A case-control study in a UK primary care setting reported an increased risk of breast cancer for patients with migraines (HR = 1.16, 95% CI = 1.09~ 1.24) [4]. Although migraines are more frequently reported among women than men, no study to date has examined the risks of malignant brain tumors among men with migraines. Our study pioneered the investigation and found an even stronger strength of association between brain tumors and a prior migraine history among men than women. Future studies need to replicate and assess possible justifications for the observed sex differences.

The underlying mechanism explaining the association between migraines and brain tumors is likely to be multifactorial, and to involve both pathophysiological processes as well as environmental circumstances. It has not been clearly addressed, yet a preliminary biological plausibility exists, involving inflammatory activities. The possibility of a continuing state of systemic or central nervous system inflammation among patients with headaches was examined. It was proposed that tumor necrosis factor (TNF)-α is a proinflammatory cytokine engaged in brain immune and inflammatory responses, as well as in pain initiation [33]. Indeed, almost all patients with new daily persistent headaches demonstrated an increase of cerebrospinal fluid (CSF) TNF-α levels, suggesting a role for TNF-α in the pathogenesis of this illness. Increased levels of serum TNF-α and interleukin (IL)-6 were also demonstrated during migraine attacks [34]. On the other hand, recent reports showed that TNF receptors, which play essential roles in inflammation and immune responses, may be involved in tumorigenesis, metastasis, and invasion by suppressing nuclear factor (NF)-jB activation [35]. As TNF receptors were found to mediate mitogenic effects in many cell types, it was proposed that the reported proliferative effects of TNF-α on astrocytes and C6 glioma cells were mediated by these receptors [36]. In addition, studies also showed that TNF-α may produce increased tumor cell growth, invasion, and progression, including gliomas (i.e., a common type of primary brain tumors) [37–39]. Future studies need to further clarify these preliminary findings connecting migraines to brain tumors possibly through inflammatory activities.

There are prominent implications of this study. As brain neoplasms are most treatable in their earlier stages [40], our results suggest to increase awareness of the possibilities of brain tumors among patients with migraines for both early detection and patient health. Appropriate adherence to screening and regular medical follow-ups after a migraine diagnosis might assist in early recognition of key symptoms of malignant brain tumors (e.g., numbness, seizures, changes in sensation, nausea, or vomiting). Proper clinical referrals and diagnostic testing could thus be prompted, together with more-aggressive management and treatment of migraines. Nevertheless, it might yet be too preliminary to disclose the potential brain tumors risk for patients with migraines as they might inappropriately and catastrophically interpret their symptoms to worsen the prognosis. We suggest that physicians and neurologists, with increased awareness of the potential risks of brain cancer, continue to monitor patients’ neurological presentation after a migraine diagnosis. Imaging of migraine patients for tumors generally is not cost effective but is necessary if profiles of symptom features suggest underlying mass lesions. These management procedures advised by our data may help enhance the chance of detecting malignant brain tumors in their earlier, most-curable stages [40].

Our findings should be interpreted cautiously due to the following limitations. First, the claims database represented patients who had sought treatment. Migraine has been considered an underdiagnosed and undertreated disease. In a study drawing participants representative of the US population, only 20% of patients who met the criteria for chronic migraine were appropriately diagnosed [41]. However, in Taiwan’s National Health Insurance (NHI) program, the characteristics of the very low out-of-pocket copayments, comprehensive benefits, unrestricted access to any medical institution of the patient’s choice, and a wide variety of providers including primary care physicians have shown to facilitate people’s utilization of healthcare. In the analyses of NHI program in 2002, only 7.7% people did not have any visit [42]. Due to the severe headache attacks that affected life quality and the traits of NHI program in Taiwan, the underdiagnosed concern of migraine may moderately be alleviated. In addition, because the healthcare utilization was recorded chronologically in this nested case-controls study, there was no apparent reason to consider that patients with and without malignant brain tumors afterwards would present distinctively on prior healthcare visits for migraine. This non-differential misclassification of exposure would possibly bias our result toward the null. Second, the diagnostic validity of migraines can be a concern. In Taiwan, migraines are generally diagnosed based upon IHS criteria in clinical settings [2].We further ensured in our study that at least one of the ≥2 migraine diagnoses (i.e., the criteria to be recruited for analyses) was delivered by a certified neurologist.

Third, the possibility of a detection or ascertainment bias cannot be ruled out. Patients with a prior migraine diagnosis may receive more medical examinations (e.g., magnetic resonance images, CT scans, etc.), leading to a higher detection rate of subsequent brain neoplasms, than non-migraine individuals. Nevertheless, the greater extent of the strength of the association observed in our study might not be well eliminated after ascertainment bias is considered. Finally, the methodology of the study is not suitable to detect a cause-effect relationship between migraine and brain tumors. Our claims dataset further lacked information on certain patient characteristics and lifestyle-related factors (e.g., stress, smoking, caffeine uptake, alcohol consumption, diet, sleep, body mass index, and family history) which could possibly compromise our findings. In the case of brain neoplasms, however, a consensus has seldom been reached as to their definite risk factors or etiology [43, 44].

## Conclusions

In conclusion, in utilizing a nationwide population-based dataset with a sizable number of patients with brain tumors, our study contributes to the previous literature by observing the occurrence of brain tumors to be associated with prior migraine diagnoses, with men presenting an even stronger strength of association than women. Although epidemiologic evidence suggests the association, replication of studies to validate migraines per se as a risk factor for brain neoplasm development is imperative, both scientifically (i.e., to characterize the potential causal link) and clinically (i.e., to direct patient management). Future studies are required to elucidate the mechanism and possible causal link, if any, connecting migraines to brain tumors, together with the potential effects of migraine treatment on the risks of consequent brain neoplasms.
